# The Immunosuppressant FTY720 (Fingolimod) enhances Glycosaminoglycan depletion in articular cartilage

**DOI:** 10.1186/1471-2474-12-279

**Published:** 2011-12-12

**Authors:** Martin H Stradner, Hannes Angerer, Thomas Ortner, Florentine C Fuerst, Daniela Setznagl, Marie-Luise Kremser, Josef Hermann, Winfried B Graninger

**Affiliations:** 1 Division of Rheumatology and Immunology, Medical University of Graz, Austria

**Keywords:** chondrocyte, fingolimod, FTY720, interleukin-1β, tumor necrosis factor-α, inducible nitric oxide synthase, glycosaminoglycan

## Abstract

**Background:**

FTY720 (Fingolimod) is a novel immunosuppressive drug investigated in clinical trials for organ transplantation and multiple sclerosis. It acts as a functional sphingosine-1-phosphate (S1P) receptor antagonist, thereby inhibiting the egress of lymphocytes from secondary lymphoid organs. As S1P is able to prevent IL-1beta induced cartilage degradation, we examined the direct impact of FTY720 on cytokine induced cartilage destruction.

**Methods:**

Bovine chondrocytes were treated with the bioactive phosphorylated form of FTY720 (FTY720-P) in combination with IL-1beta or TNF-alpha. Expression of MMP-1,-3.-13, iNOS and ADAMTS-4,-5 and COX-2 was evaluated using quantitative real-time PCR and western blot. Glycosaminoglycan depletion from cartilage explants was determined using a 1,9-dimethylene blue assay and safranin O staining.

**Results:**

FTY720-P significantly reduced IL-1beta and TNF-alpha induced expression of iNOS. In contrast FTY720-P increased MMP-3 and ADAMTS-5 mRNA expression. Furthermore depletion of glycosaminoglycan from cartilage explants by IL-1beta and TNF-alpha was significantly enhanced by FTY720-P in an MMP-3 dependent manner.

**Conclusions:**

Our results suggest that FTY720 may enhance cartilage degradation in pro-inflammatory environment.

## Background

Since the introduction of the anti-TNF antibody as a therapy for rheumatoid arthritis (RA) in the late 1990's the armamentarium of protein-based immune-modulating drugs has steadily increased. In the next decade rheumatologists will be able to apply a novel class of therapeutic tools. The new group of small-molecule drugs are specific inhibitors of inter- or intra-cellular signalling pathways. They are orally active and can be produced at low cost. However, the target molecules of these drugs may be expressed in other organ systems as well. This may result in unforeseeable adverse events upon long-time treatment.

FTY720 (fingolimod) is such a novel small-molecule immunosuppressant. Its immunosuppressive efficacy has been documented in large-scale prospective phase III studies in renal transplantation [1] and multiple sclerosis [2,3]. Oral intake of FTY720 leads to marked reduction of the number of circulating T- and B lymphocytes [1]. Unlike other immunosuppressants it does not impair lymphocyte proliferation, nor does it induce apoptosis of lymphocytes [4].

Due to its structural analogy with the naturally occurring lipid sphingosine most cells take up FTY720 to phosphorylate it to its bioactive form FTY720-P [5]. In lymphocytes FTY720-P interacts with the receptors for Sphingosine-1-phosphate (S1P), thereby internalizing the S1P receptor-subtype 1 (S1P_1_) [6]. Since the S1P_1 _receptor is necessary to egress the lymph nodes, its neutralization inhibits the migration of lymphocytes into the circulation leading to the immunosuppressive activity of this substance [7].

While clinical trials of FTY720 in rheumatic diseases have not been registered so far, its use in RA has been proposed [8]. In rat collagen-induced arthritis FTY720 inhibited the formation of synovitis and bone erosions more effectively than prednisone [9]. In the adjuvant-induced arthritis rat model FTY720 inhibited joint inflammation as successful as tacrolimus and cyclosporin A [10].

Moreover, FTY720 may also impair S1P signaling in other cell types. For chondrocytes S1P is protective by limiting glycosaminoglycan (GAG) degradation and inducing proliferation [11,12]; moreover it stimulates prostaglandin E2 release via S1P receptor subtypes S1P_1-3 _[13]. Direct effects of FTY720 on articular cartilage have not been reported so far. Since FTY720 leads to a perturbation of S1P signaling, its use as an immunosuppressant could have an impact on articular cartilage.

This study therefore aims to evaluate the in vitro impact of FTY720 on chondrocytes in the presence of pro-inflammatory cytokines in analogy to the situation found within rheumatoid arthritis joints. As risk indicators for cartilage damage we used *iNOS *expression and release of GAG.

## Methods

### Reagents

FTY720-P was kindly donated by Novartis Pharma AG (Basel, Switzerland) and dissolved in DMSO-HCL. Bovine IL-1β and TNF-α were purchased from AbD Serotec(Oxford, UK). XG076 was purchased at Calbiochem (Darmstadt, Germany). DMEM High Glucose with L-Glutamine, DMEM/Ham's F-12 with L-Glutamine 1:1, FCS and Penicillin/Streptomycin solution was purchased at PAA Laboratories (Pasching, Austria). We acquired iNOS antibodies (Upstate, Lake Placid, NY) and antibodies for actin (Sigma-Aldrich, St. Louis, MO). Secondary antibody was purchased from Cell Signaling (Danvers, MA).

### Cell culture

Cartilage was harvested from bovine metacarpophalangeal joints of adult cows (20 - 24 months, n = 21) under aseptic conditions. Cartilage tissue was minced and digested in 0.2% collagenase B (F. Hoffman La Roche Ltd., Basel, Switzerland) for 16 hours. The resulting cell suspension was filtered through a nylon mesh with pores of 70 μm (BD Pharma, Le Pont-De-Claix, France). Cells were counted and viability tested using trypan blue dye (Sigma-Aldrich). Bovine chondrocytes were then cultured in monolayer at 37°C, 5% O2 and 5% CO2, in DMEM/F-12 1:1 supplemented with 10% FCS and 1% Penicillin/Streptomycin solution over 1 passage. Upon 80%-90% confluence cultures were incubated with serum free medium 24 h prior to experiments. Chondrocyte viability in the presence of 0.01 μM to 10 μM FTY720-P, the corresponding amount of vehicle (DMSO-HCL) and 100 μM XG076 was assessed for 72 h using a MTS ([3-(4,5-dimethylthiazol-2-yl)-5-(3-carboxymethoxyphenyl)-2-(4-sulfophenyl)-2H-tetrazolium) assay (Promega, Madison, WI) according to the manufacturers protocol. MTS assay was carried out in hexaplicates and repeated three times using chondrocytes from 3 different animals

### Real-time PCR

Three independent experiments using chondrocytes from 3 different animals were performed in duplicates for each treatment group. Chondrocytes were allowed to grow over one passage and upon 80%-90% confluence cultures were serum-depleted 24 h prior to experiments. Chondrocytes were then treated with 10 ng/ml IL-1β or 100 ng/ml TNF-α in combination with 0.1 to 3 μM FTY720-P or vehicle solution for 3 h. RNA was isolated using Tri-reagent (Sigma-Aldrich, St. Louis, MO) according to the manufacturers recommended protocol. To avoid potential DNA contamination, DNA was digested with 1 U DNase (Fermentas, Burlington, ON) per μg RNA for 30 minutes at 37°C. A total of 1 μg of RNA was then reverse transcribed using RevertAid cDNA Synthesis Kit (Fermentas) and random hexamer primers according to the provided manual. For amplification all samples were run in triplicates using a master mix containing SYBR green (2× SYBR Green PCR Master Mix, Invitrogen Corp., Carlsbad, CA). Primers (MWG, Ebersberg, Germany) were designed with Primer3 software [14]. Sequences of primers are listed in additional file 1, Table S1. For normalization the housekeeping genes Hydroxymethyl-bilane synthase, Beta-2-microglobulin, Beta actin, Hypoxanthine phosphoribosyltransferase 1, Ribosomal protein S18 and Glyceraldehyde-3-phosphate dehydrogenase (GAPDH) were evaluated in our experimental setting. GAPDH showed the least variation over all treatments and was therefore chosen as housekeeping gene for the following analyses. The relative amount of cDNA was calculated using ABI prism sequence detecting software (Applied Biosystems) based on a standard curve, according to the manufacturers' manual. Minimum information for publication of quantitative real-time PCR experiments is supplied in additional files 1 and 2[15].

### Western blot analysis

Three independent experiments using chondrocytes from 3 different animals were performed. Chondrocytes were allowed to grow over one passage and upon 80%-90% confluence cultures were serum-depleted 24 h prior to experiments. Total protein was extracted from cultured chondrocytes after 24 h of treatment with10 ng/ml IL-1β in combination with 3 μM FTY720-P or vehicle solution, using lysis buffer (50 mM Tris-HCL, 4.1 M NaCl, 0.1% Triton X-100, 5 mM EDTA, 1% protease inhibitor cocktail; all Sigma Aldrich). After brief sonication protein concentration was measured with DC Protein assay (BioRad). 7 μg of protein were separated by SDS-PAGE using a 10% polyacrylamide gel and were transferred to a nitrocellulose membrane (BioRad, Hercules, CA). After overnight blocking in 5% skim milk in Tris-buffered saline the membranes were incubated with primary antibodies against iNOS (1:2000) and actin (1:5000) for 2 h. Thereafter membranes were rinsed in blocking solution and incubated for 1 h with secondary antibody conjugated to horseradish peroxidase (1:3000). Bands were visualized using ECL plus detection system (Amersham, Arlington Heights, IL).

### Determination of Glycosaminoglycan (GAG) release

Three independent experiments were performed using cartilage from 3 different animals. Cartilage explants of 4 × 4 × 2 mm (wet weight 13,3 mg ± 0.9 mg) were harvested from bovine metacarpophalangeal joints under aseptic conditions and cultured at 37°C, 5% O2 and 5% CO2, in DMEM/F-12 1:1 supplemented with 10% FCS and 1% Penicillin/Streptomycin solution for 24 h. After another 24 h in the absence of FCS cartilage explants were treated with 0.5-3 μM FTY720-P or vehicle control and 10 ng/ml IL-1β or 100 ng/ml TNF-α over 5 days. Medium was changed and treatment renewed on day 3. Treatment was carried out in triplicates for each treatment group. Thereafter explants were weighted and digested in phosphate buffer (2.76 g/l NaH2PO4, 0.38 g/l EDTA, 0.25 g/l DTT all Sigma-Aldrich, pH 6.8) containing 0.6 g/l papain (Sigma-Aldrich). GAG concentration in culture supernatants of day 3, day 5 and the digested explants was determined by 1,9-dimethylene blue (DMB; Sigma-Aldrich) reaction [11,16]. In brief 20 μl of the samples and chondroitin sulphate (Sigma-Aldrich) standard dilution were incubated with 180 μl DMB solution (38.5 μM DMB, 40 mM NaCl, 40 mM glycin, 0.5% Ethanol, pH 3) for 5 minutes. Absorbance at 530 nm was measured with Spectra max 384-plate reader. Results were normalized to wet weight of the explants.

### Histological examination

Three independent experiments using cartilage from 3 different animals were performed. Bovine cartilage *e*xplants were harvested as described above and were cultured at 37°C, 5% O2 and 5% CO2, in DMEM/F-12 1:1 supplemented with 1%Penicilin/Streptomycin solution. Media containing either 10 ng/ml IL-1β or 100 ng/ml TNF-α in combination with 3 μM FTY720-P or vehicle solution was changed on the 3^rd ^and 6^th ^day. Upon 8 days of treatment, cartilage explants were fixed in 4% formalin solution for 24 hours and then embedded in paraffin. Sections of 3 μm were stained with 0.1% safranin O solution, 0.001% fast green solution and Weigert's iron hematoxylin solution [17].

### Statistical analysis

Results are presented as mean ± standard error of the mean (SEM). Real-time RT-PCR results are expressed as mean ± SEM percentage over control. Normal distribution of the data was assessed using Kolmogorov-Smirnov test. Data in all treatment groups were normally distributed. Data were analyzed by one-way ANOVA followed by post-hoc analysis using Bonferroni corrected t-test. SPSS 13.0 (IBM, Chicago, IL) was used for statistical analysis. Differences between two groups were considered significant at the P < 0.05 level.

## Results

### FTY720-P reduces cytokine induced iNOS expression

**Figure 1:**
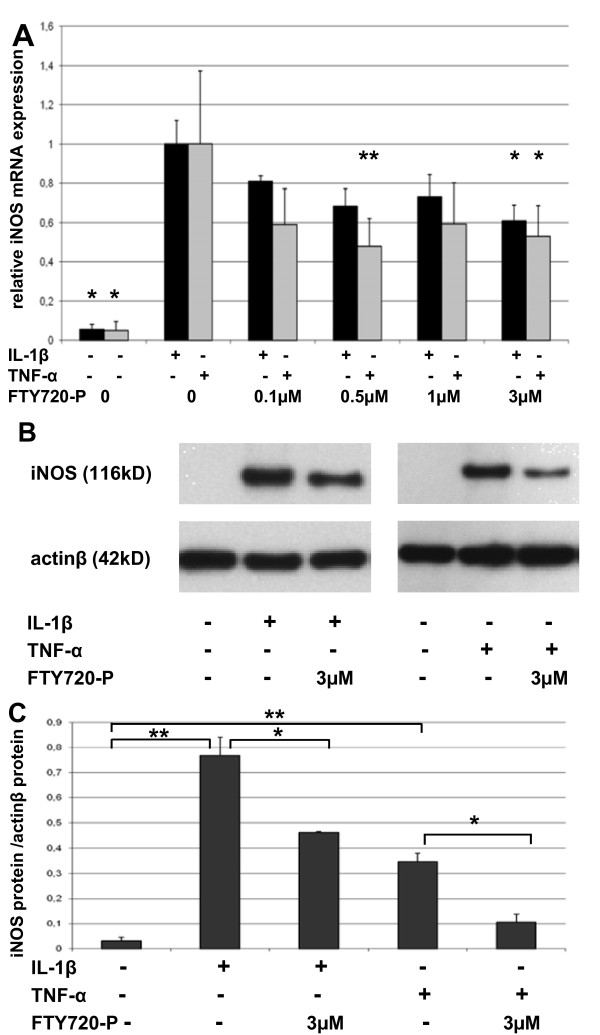
**FTY720-P reduces cytokine-induced iNOS expression**. (A) *iNOS *mRNA was quantified by real-time PCR after 3 h of treatment of chondrocytes grown in monolayer with the indicated concentrations of FTY720-P in combination with 10 ng/ml IL-1β or 100 ng/ml TNF-α. Values were normalized to *GAPDH *and are presented as percentage of *iNOS *expression induced by cytokine treatment. Data are means ± SEM of 3 independent experiments. *P < 0.05 versus cytokine treated vehicle control. (B) Representative Western blot from total protein isolated after 24 h treatment with 10 ng/ml IL-1β or 100 ng/ml TNFα and co-treatment with 3 μM of FTY720-P or the appropriate amount of vehicle solution (C) Intensity of Western blots was quantified by densitometry and is expressed as a ratio of *iNOS *versus actin. Data are means ± SEM of 3 independent experiments. *P < 0.05, ** *P *< 0.01.

Chondrocyte viability was unchanged in the presence of up to 10 μM FTY720-P or 100 μM of the MMP-3 inhibitor XG076 (P > 0.05, data not shown). Treatment of chondrocytes with 10 ng/ml interleukin-1 (IL-1)β resulted in 18-fold up-regulation of *iNOS *mRNA expression (P < 0.05). Co-treatment with up to 3 μM FTY720-P reduced iNOS mRNA expression by 40 ± 8% (mean ± SEM, *P *< 0.05) compared to vehicle solution. (Figure 1A)

Treatment with 100 ng/ml tumor necrosis factor (TNF)-α led to a 18-fold (*P *< 0.05) up-regulation of *iNOS *mRNA. Co-treatment with 3 μM of FTY720-P reduced TNF-α induced *iNOS *expression by 47 ± 16% (*P *< 0.05). A similar trend was observed when IL-1β stimulated chondrocytes were co-treated with unphosphorylated FTY720 (data not shown). The maximal effect was observed later (after 6 h) and was less pronounced compared to treatment with FTY720-P.

iNOS protein expression was significantly enhanced after 24 h of treatment with IL-1β (24-fold, *P *< 0.01) or TNF-α (11-fold, *P *< 0.01). As expected from RT-PCR results, co-treatment with 3 μM of FTY720-P reduced IL-1β and TNF-α induced iNOS protein expression by 39%, (*P *< 0.05) and 69.4% (*P *< 0.05) respectively (Figure 1B and 1C).

### FTY720-P enhances cytokine induced glucosaminoglycan (GAG) depletion

**Figure 2:**
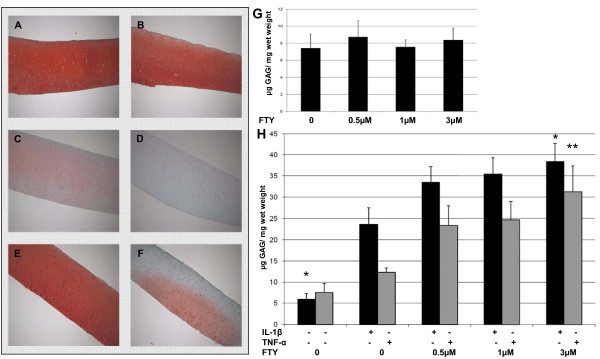
**FTY720-P enhances cytokine-induced GAG loss**. Cartilage explants were treated as indicated for 8 days. Formalin fixed and paraffin embedded explants were then stained with safranin O. (A) untreated; (B) 3 μM FTY720-P; (C) 10 ng/ml IL-1β; (D) 10 ng/ml IL-1β and 3 μM FTY720-P; (E) 100 ng/ml TNF-α; (F) 100 ng/ml TNF-α and 3 μM FTY720-P. The figure shows representative pictures taken from one of 3 independent experiments. (G) GAG depletion was assessed by 1,9-dimethylene blue assay after treatment over 5 days with FTY720-P alone or (H) in the presence of 10 ng/ml IL-1β and 100 ng/ml TNF-α. Data are means ± SEM of 3 independent experiments. *P < 0.05, ** *P *< 0.01.

Culturing cartilage explants for 8 days with 0.1-3 μM of FTY720-P did not result in altered depletion of GAG compared to vehicle treated cartilage (Figure 2G). IL-1β induced a significant increase in GAG depletion (Figure 2H). Untreated cartilage explants lost 5.9 ± 1.3 μg GAG/mg wet weight over 5 days compared to 23.7 ± 3.9 μg/mg lost by IL-1β treated explants (*P *< 0.05). Furthermore co-treatment with FTY720-P resulted in significantly higher amounts of GAG loss. Co-treatment of IL-1β with 3 μM of FTY720 increased GAG depletion to 38.4 ± 4.3 μg/mg (*P *< 0.05 compared to IL-1 treatment). Treating cartilage explants with TNF-α led to a GAG depletion of 12.3 ± 1.0 μg/mg, which was not significantly different from untreated explants (7.5 ± 2.1 μg/mg, *P *= 0.18). Upon incubation with FTY720-P a significant increase in GAG depletion was observed. 3 μM of FTY720-P led to 31.2 ± 6.2 μg/mg (*P *< 0.01 vs. TNF-α) of GAG depleted from cartilage explants (Figure 2H).

These results were confirmed by histology in an independent series of experiments. GAG content as determined by safraninO staining in FTY720-P treated cartilage was not different from untreated cartilage (Figure 2A and 2B). IL-1β treatment resulted in considerable discoloration of cartilage. Co-treatment with 3 μM of FTY720-P further enhanced the effect (Figure 2C and 2D). Again TNF-α treatment showed a mild negative effect on cartilage staining. Combination with FTY720-P led to discoloration of the upper half of the cartilage explants. This effect was independent of whether cartilage explants were placed in the culture wells correctly or up-side down (Figure 2E and 2F).

### FTY720-P mediated GAG depletion is dependent on functional MMP-3

**Figure 3:**
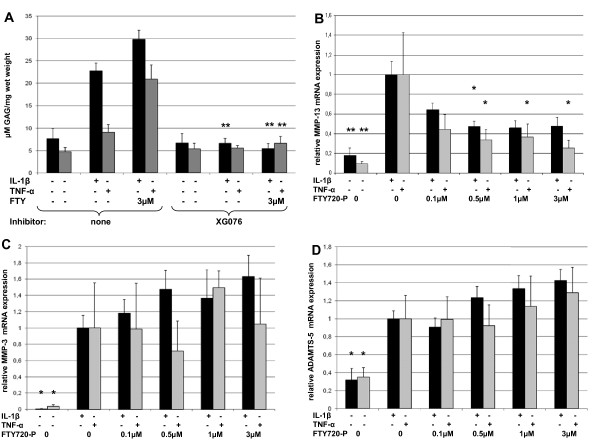
**GAG depletion is dependent on MMP-3 activation**. (A) GAG depletion was assessed by 1,9-dimethylene blue assay after treatment for 5 days in the presence of 100 μM XG076. Data are means ± SEM of 3 independent experiments. *P < 0.05, **P < 0.01 vs. corresponding treatment without inhibitor. (B) *MMP*-13, (C) *MMP*-3 and (D) *ADAMTS*-5 mRNA was quantified by real-time PCR after 3 h of treatment with the indicated concentrations of FTY720-P in combination with 10 ng/ml IL-1β or 100 ng/ml TNF-α. Values were normalized to *GAPDH *and are presented as percentage of gene expression induced by cytokine treatment. Data are means ± SEM of 3 independent experiments. *P < 0.05, ** *P *< 0.01 versus cytokine treated vehicle control.

As *MMP*-3 is crucial for activation of pro-MMP's, we used XG076, an inhibitor of pro-MMP-3 activation. Upon inhibition of pro-MMP-3 with XG076 the effect of IL-1β and TNF-α on GAG depletion was rendered to levels of the untreated controls. Furthermore co-treatment with 3 μM FTY720-P did not result in increased GAG depletion (Figure 3A).

### FTY720-P reduces cytokine-induced MMP-13, but enhances MMP-3 and ADAMTS-5 expression

To asses which protease was responsible for the enhanced GAG depletion by FTY720-P we examined gene expression of *MMP*-1,-3.-13, *ADAMTS*-4,-5 and COX-2. Treatment of chondrocytes with either IL-1β or TNF-α led to a 5.5-fold and 8.4-fold up-regulation of *MMP*-13 mRNA, respectively (both with *P *< 0.01). Co-treatment with FTY720-P reduced *MMP*-13 mRNA expression by 52 ± 8% and 74 ± 9% (both *P *< 0.05, Figure 3B)

In contrast to the effect of FTY720-P on *MMP*-13, *MMP*-3 and ADAMTS-5 expression induced by IL-1β was further enhanced by FTY720-P, however not reaching statistical significance (*P *= 0.063 and 0.054 respectively; Figure 3C and 3D). Expression of COX-2, *ADAMTS*-4 and *MMP*-1 was enhanced by TNF-α and IL-1β treatment. No effect was observed upon co-treatment with FTY720-P (data not shown).

## Discussion

Our study examines the impact of the small-molecule immunosuppressant FTY720 on articular cartilage. We demonstrate that FTY720-P strongly enhances GAG degradation in the presence of the inflammatory cytokines IL-1β and TNF-α. This effect may result from FTY720's functional antagonism on S1P signaling. FTY720-P leads to internalization of the S1P_1 _receptor thereby compromising S1P signaling [6]. S1P however physiologically protects articular cartilage from IL-1β induced GAG loss [11]. Interestingly FTY720-P does not lead to GAG loss in the absence of cytokines, suggesting that either activation of catabolic signaling pathways or the occurrence of cartilage damage is required by FTY720-P to inflict GAG depletion. The latter is supported by the finding that FTY720-P failed to induce GAG loss in the presence of inflammatory cytokines when their degradative effect was blocked by XG076.One can hypothesize a negative feed-back loop induced by active MMP's or degraded GAG involving the S1P_1 _receptor, in order to confine uncontrolled cartilage destruction. Internalization of S1P_1 _by FTY720-P would block such a pathway resulting in enhanced GAG destruction by IL-1β and TNF-α.

In order to identify the proteases responsible for the break-down of GAG, we investigated the impact of FTY720-P co-treatment on the expression of genes implicated in GAG metabolism. We did not find a correspondent change of expression in the genes examined. The trend towards increase of *MMP*-3 and *ADAMTS*-5 expression upon co-treatment of FTY720-P with IL-1β is unlikely to cause the pronounced effect on GAG metabolism. Therefore other proteases or post-transcriptional mechanisms may be responsible for the enhanced loss of GAG.

In animal models of rheumatoid arthritis administration of FTY720 prevented or ameliorated joint inflammation due to its immunosuppressive properties [9,10,18]. None of these studies investigated cartilage break-down in detail. The persistence of cartilage damage in spite of FTY720 treatment was reported in SKG mice [18]. However, the study design did not allow the assessment of a possible direct impact of FTY720 on cartilage damage. Interestingly, FTY720 was not able to alter the course of collagen-induced arthritis when it was administered after the disease had developed [19]. In this setting there was a trend of increased joint damage in mice treated with FTY720 compared to untreated mice.

Contrary to the effects of FTY720-P on GAG metabolism *iNOS *and *MMP*-13 gene expression were significantly diminished by co-treatment with FTY720-P. This effect was also observed in the absence of TNFα or IL-1β (data not shown) and is similar to chondrocytes treated with S1P [11]. It may therefore result from the agonistic impact of FTY720-P on S1P_3 _receptors [20]. In line with these findings a previous study reported that S1P diminished IL-1 induced iNOS expression in renal mesangial cells [21]. In cartilage excess of nitric oxide (NO) produced by iNOS forces chondrocytes to undergo apoptosis [22,23]. Furthermore, NO inhibits GAG and collagen II synthesis in chondrocytes and iNOS knock-out mice exhibit less cartilage degradation in models of OA and RA than their wild-type litter mates [24-26]. Reduction of iNOS expression by S1P and FTY720-P may inhibit excess of NO thereby allowing normal production of extra-cellular matrix constituents. In our experimental setting this potentially anabolic effect could not compensate for the catabolic effect of FTY720-P on GAG homeostasis.

One limitation of the current study is the use of bovine chondrocytes. Furthermore part of the data was obtained from chondrocytes in monolayer culture. Moreover, it is unknown if concentrations of FTY720-P used in this study are found in human joints. In an animal study Matsumoto et al. measured a mean serum concentration of 0.1 μM FTY720 after oral administration of 1 mg/kg to rats [27]. Assuming that similar concentrations are present in synovial fluid, the concentrations of FTY720-P used in the current study would exceed FTY720 levels in rats ten-fold. This however seems acceptable as FTY720 is phosphorylated intracellularly and released to act in an auto- and paracrine manner. Therefore local concentrations of FTY720-P may well exceed those of systemic FTY720.

## Conclusions

Our data indicate that FTY720-P influences the metabolism of chondrocytes in a dichotomous way. As an agonist of the S1P_3 _receptor FTY720-P reduces expression of *iNOS *and *MMP*-13, whereas it enhances GAG destruction probably due to its antagonistic properties at the S1P_1 _receptor. Therefore the administration of FTY720 might enhance loss of cartilage in an inflammatory environment. Especially in pre-existing osteoarthritis and in rheumatoid arthritis with abundant expression of TNF-α and IL-1β the use of FTY720 may accelerate cartilage degradation. However, further research is needed to elucidate the impact of FTY720 on cartilage in human joint diseases.

## List of abbreviations

ADAMTS: a disintegrin and metalloproteinase with thrombospondin motifs; COX: cyclooxygenase; DMB: 1,9-dimethylene blue; DMSO: Dimethyl sulfoxide; FTY720-P: phosphorylated form of FTY720; GAG: glycosaminoglycan; *GAPDH: *glyceraldehyde 3-phosphate dehydrogenase;IL-1: interleukin-1; *iNOS: *inducible nitric oxide synthase; MMP: matrixmetaloprotease; RA: rheumatoid arthritis; S1P: sphingosine-1-phosphate; S1P_1-5_: S1P receptor sub-type 1 to 5; SEM: standard error of the mean; TNF-α: tumor necrosis factor α.

## Declaration of competing interests

The authors declare that they have no competing interests.

## Authors' contributions

MS participated in the design of the study, carried out the data analysis and drafted the manuscript. HA participated in the design of the study and carried out the analysis of GAG release. TO participated in the design and helped to draft the manuscript. FF participated in the design and helped to draft the manuscript. DS did the real-time PCR and western blot studies and helped to draft the manuscript. MK carried out the histology and helped to draft the manuscript. JH participated in the design of the study and helped to draft the manuscript. WG participated in the design of the study and helped to draft the manuscript. All authors read an approved the final manuscript.

## Pre-publication history

The pre-publication history for this paper can be accessed here:

http://www.biomedcentral.com/1471-2474/12/279/prepub

## Supplementary Material

Additional file 1 **Table S1**. Primers and protocol of real-time PCR. Click here for file

Additional file 2 **Table S2**. Minimum information for publication of quantitative real-time PCR experiments. Click here for file
