# Supplementary material for: The Immunosuppressant FTY720 (Fingolimod) enhances Glycosaminoglycan depletion in articular cartilage
Source: BMC Musculoskelet Disord. 2011 Dec 12;12:279. doi: 10.1186/1471-2474-12-279 (PMC3258222; doi:10.1186/1471-2474-12-279)
Supplement: Additional file 1 — Table S1. Primers and protocol of real-time PCR. [file 1471-2474-12-279-S1.DOCX]

Additional file 1

Table S1

| Gene name | Accession number | Exon location | Amplicon size | | Sequence forward | Sequence reverse | | PCR efficacy (%) | Limit of detection (C_t_) |
| --- | --- | --- | --- | --- | --- | --- | --- | --- | --- |
| ADAMTS-4 | NM_181667.1 | 4/5 | 218bp | | 5´-GAAGCAATGCACTGGTCTGA-3 | 5´-GGTCAGCGTCGTAGTCCTTG-3´ | | 97.3 | 33 |
| ADAMTS-5 | NM_001166515.1 | 6/7 | 121bp | | 5´-ccaaatatgctggtgtcctg-3´ | 5´-ctgtatggcctgcactctgt-3´ | | 108.6 | 32 |
| COX-2 | NM_174445.2 | 10 | 264bp | | 5´-tccatgtcagaatcgaggtg-3´ | 5´-agtgcactgtgttgggagtg-3´ | | 88.8 | 33.5 |
| GAPDH | NM_001034034.1 | 5/8 | 299bp | | 5´-ggtgatgctggtgctgagta -3´ | 5´-gtcttctgggtggcagtgat-3´ | | 94.3 | 34.5 |
| MMP-1 | NM_174112.1 | 4/7 | 396bp | | 5´-TGCTCATGCTTTTCAACCAG-3´ | 5´-TCCACTTCTGGGTACAAGGG-3´ | | 89.6 | 35 |
| MMP-3 | NM_001206637.1 | 5 | 109bp | | 5´-CACTCAACCGAACGTGAAGCT-3´ | 5´-CGTACAGGAACTGAATGCCGT-3´ | | 112.3 | 32 |
| MMP-13 | NM_174389.2 | 2/3 | 202bp | | 5´-ggtgactggcagacttgatg-3´ | 5´-ccacaccttgaaggcttttc-3´ | | 102.8% | 36.5 |
| Protocol | | | | | | | | | |
| 10µl 2XMaster Mix, 1µl (4pM) fw. Primer, 1µl (4pM) rev.Primer,3µl distilled water, 5µl (25ng) cDNA | | | | | | | | | |
| Pre-amplification: 2’ 50°, 2’ 95° | | | | **Amplification:** 40 cycles: 15’’95°, 30’’60° | | | **Dissociation:**15’’60°, 15’’95° - Dissociation Stage - 30° | | |
