# Supplementary material for: The Immunosuppressant FTY720 (Fingolimod) enhances Glycosaminoglycan depletion in articular cartilage
Source: BMC Musculoskelet Disord. 2011 Dec 12;12:279. doi: 10.1186/1471-2474-12-279 (PMC3258222; doi:10.1186/1471-2474-12-279)
Supplement: Additional file 2 — Table S2. Minimum information for publication of quantitative real-time PCR experiments. [file 1471-2474-12-279-S2.DOCX]

Additional file2

Table S2

| **Sample/Template** | **details** |
| --- | --- |
| Source | Chondrocytes cultured in monolayer |
| Method of preservation | No preservation |
| Storage time (if appropriate) | none |
| Handling | fresh |
| Extraction method | TriZol |
| RNA: DNA-free | Intron-spanning primers when possible/DNA digest |
| Concentration | none |
| RNA: integrity | Spectrophotometry |
| Inhibition-free | Sample dilution |
| **Assay optimisation/validation** |  |
| Accession number | See additional file 1, Table S1 |
| Amplicon details | See additional file 1, Table S1 |
| Primer sequence | See additional file 1, Table S1 |
| *Probe sequence** | None (SYBR Green) |
| *In silico* | Primer3 software, Primer-BLAST checked |
| empirical | primer concentration 4pM/annealing temperature 60°C |
| Priming conditions | random |
| PCR efficiency | See additional file 1, Table S1 |
| Linear dynamic range | 5 Log |
| Limits of detection | See additional file 1, Table S1 |
| Intra-assay variation | Not tested (one operator, one site) |
| **RT/PCR** |  |
| Protocols | See additional file 1, Table S1 |
| Reagents | See Material&Methods section |
| Duplicate RT | ΔCq<0.5 |
| NTC | Cq>39, no melt curve peek |
| NAC | none (SYBR Green) |
| Positive control | calibration curve in every run |
| **Data analysis** |  |
| Specialist software | ABI prism sequence detecting software |
| Statistical justification | Duplicate treatment in 3 independent experiments using 3 different animals each. |
| Transparent, validated normalisation | See Methods section |
